# Fœticide

**Published:** 1857-01

**Authors:** J. Boring

**Affiliations:** Professor Obstetrics, &c., Atlanta Medical College


ATLANTA

Medical and Surgical Journal.

VOL. II.] JANUARY, 1857. [NO. 5.

### ORIGINAL COMMUNICATIONS.

*Fœticide.* An Essay by J. BORING, M. D., Professor Obstetrics, &c., Atlanta Medical College; read before the Atlanta Medical Society, and published by that body.

In the selection of a subject for discussion on the present occasion, I have been governed by a desire for the advancement of medical science, and sound morality.

Fœticide has been chosen from a settled conviction that its nature and importance are not generally appreciated by mankind, and that some method should be adopted by which public attention and investigation may be had.

The opinion, so long and generally entertained, that the fœtus in utero *becomes* a living being at birth, or, at least, at the time of "quickening," is, in my judgment, based upon profound error, and productive of consequences the most painful and revolting to the enlightened Christian mind.

It has, in innumerable instances, lead the unsuspecting mother to the horrid crime of shedding the blood of her own innocent offspring, and made her husband accessory to the unnatural cruelty.

It has presented an open door of escape from punitive justice, to the multitudes of fallen women, whose passions and foul commerce have subjected them to illegal pregnancy.

It has covered the depravity of scores of those in what is called "high life," and who, but for the hope of escape from exposure by means of criminal abortion, would probably never have fallen by the snares of the seducer.

In few words, it has conduced to evil, and only to evil, in that it has served to render illicit intercourse of the sexes both easy and comparatively safe from public scrutiny, while, at the same time, it has lowered the estimate of human life, and obscured the turpitude of murder.

It strikes at the very foundations of society, is reckless of virtue, and tends, in all its influences, to the destruction of conjugal integrity.

Every member of the profession knows with what apparent confidence and boldness, not the unfortunate only, who have been deceived and ensnared by the seducer, ask to be relieved from the consequences of their illegal commerce, by professional skill in superinducing abortion, but, that the virtuous and the intelligent wife and mother seek, by the adoption of the same measures, to escape the pangs of parturition, and the seclusion of the season of nursing.

Do these last know, does the physician who interposes his skill remember, that the thing proposed is cold, deliberate murder?

That there are those who recklessly resort to abortion to cover their depravity, and that there are monsters in human form, (they can hardly be regarded *as human,*) wearing, most unfortunately, the title of M. D., who, *for money*, will commit the atrocious crime, is too manifest to admit of a question; but, that very many are lead into it by ignorance of its nature and magnitude, is firmly believed. It cannot be, that the multitudes of women who are wives and mothers, and men who are husbands and fathers, who adopt this mode of evading the results of the laws by which they are bound together, do so understandingly.

They do not, as is certainly true, believe themselves murderers, and that of their own offspring. But, that every abortion induced by such causes, and prompted by such motives, is, in the sight of God, murder *out right*, seems to me not to admit of the slightest doubt.

It is unnecessary to adduce evidence of the fact that the destruction of the fœtus in utero has not been, and is not now regarded as murder, since it is true, that England and the United States, the most enlightened nations of the earth, by express statutes, only hold it to be a "high misdemeanor;"

and even *Georgia*, our own State of boasted wisdom and virtue, is found in the same category.

Is it not singular enough that one of our statutes should define murder to be "the unlawful killing of a human being in the peace of the State, with malice afore-thought, either implied or express," and that another should define the cold blooded, deliberate destruction of the fœtus in utero, to be only "a high misdemeanor?" If I am not wholly mistaken, it will be seen that, of all the varieties of murder, that of the embryonic human being is the most atrocious and indefensible. It is a wanton, unprovoked and cruel deprivation of a human being, of the existence which God alone gave, and can of right, take away, and that being is not only inoffensive but utterly helpless.

The doctrine which I hold, and now propose to establish is, that from the moment of the fecundation of an ovum, it is, in every essential sense, a living, self-sustaining, and self-developing being, entitled to protection in its possessions, and that whatever it may become, physically, mentally, and spiritually, *in* utero, or *ex*tra-utero, is, by growth and development of the original, and not by the addition of *new* materials, or attributes, and, therefore, its destruction, its wanton destruction, is alike murder, at any and every stage of its existence; and no matter whether the killing is by means of a rifle ball, a bowie-knife, a dose of ergot, or the introduction of a bougie into the uterus—the means employed, and the time chosen, to perpetuate the deed, can never change its turpitude—*it is murder still.*

Before introducing proof in support of this view, it will not be wholly irrelevant, to refer briefly to some of the numerous doctrines which have been, and are still held, by those whose position and practice are here controverted, especially, as the doing so will serve to demonstrate the folly of an attempt to settle upon some period after fecundation, at which the ovum takes on the attributes of a living being.

On this subject, Beck, in his work on Medical Jurisprudence, has the following judicious remarks: "The ancients were by far the most extravagant in their notions on this subject. The same fundamental error, however, pervaded all their doctrines. They believed that the sentient and vital principle was not infused into the fœtus until sometime after conception had taken place. It is not surprising that the exact time at which this union is effected, could never be satisfactorily settled by them. According to Hippocrates, the male fœtus became animated in thirty days after conception, while the female required forty-two.

"The stoics believed that the soul was not united to the body, before the act of respiration, and consequently, that the fœtus was inanimate during the whole period of uterogestation.

"This doctrine prevailed until the reigns ot Antoneus and Sevenes, when it gave way to the more popular sentiments of the sect of the Academy, who maintained that the fœtus became animated at a certain period of gestation.

"The common law of the Church of Rome also distinguished between the animate and inanimate fœtus, and punished the destruction of the former with the same severity as that of homicide.

"Galen considers the animation of the fœtus to take place on the fortieth day after conception; at the same time that he supposed the fœtus to become organized.

"Others believed shorter periods sufficient, and, accordingly, three days, and seven, have respectively had their advocates.

"Another contends that eighty days are requisite for the animation of the female, while only forty are necessary for the male.

"Some advocate forty days, as sufficient for both.

"Others, again, make a distinction between the imperfect embryo and the perfectly formed fœtus, and consider abortion of the latter as a crime deserving the same punishment as homicide.

"Amidst these discordant sentiments, Zachias offers himself as a mediator, and proposes sixty days as the limit, and recommends that any one who should cause an abortion after that period, whether of male or female, should be punished for homicide."

From the foregoing, it is obvious that the confusion by which the subject has been constantly encompassed, has arisen from the attempt to fix upon some period, subsequent to conception or fecundation, at which vitality is communicated to the fœtus in utero, and there being, manifestly, no such period, a satisfactory settlement of the question as to *what* time it occurs, can never be had. The whole attempt is an egregious blunder, having for its origin erroneous physiological doctrines.

It is a matter of no ordinary surprise, that both England and this country should have adopted principles so manifestly absurd and dangerous to the good of society, and thus perpetuate one of the greatest errors of Pagan nations and Romanists, and our surprise is enhanced by the considerations of life, and the tremendous responsibilities attending its criminal destruction.

On this subject, Beck says, "The absurdity of the principles on which these distinctions are founded, is of easy demonstration. The fœtus, previous to the time of quickening, must be either dead or alive. Now, that it is not the former, is most evident from neither putrefaction nor decomposition taking place, which would be the inevitable consequences of an extinction of the vital principle.

"To say that the connexion with the mother prevents this, is wholly untenable; facts are opposed to it. Fœtuses do actually die in the uterus before quickening, and then all the signs of death are present. The embryo, therefore, before that crisis, must be in a state different from that of death: and this can be no other than life."

But, the view of the case which I would present, and which appears to me the only rational one, is, that the fecundated or fertilized ovum is from the moment of its fecundation, a *bona fide human being in embryo*, and henceforward, in process of development, identically the same from its embryonic state, through uterogestation, at birth, during lactation, dentition, and manhood.

That this view is correct, will, it is believed, be sustained by all the reliable works on the subject of Physiology. Let us examine a few, only, of the most approved European and American authors.

Richerand says, "Blood is perceived about the seventeenth day after conception, together with the pulsation of the heart, and not long after the different organs have commenced their development."

Blumenbach and Magendie confirm this statement

Mauriceau states that he "saw a fœtus of about ten weeks, that was alive, move its arms and legs, and open its mouth."

Carpenter remarks, that "a change in the type of the circulating system of the fœtus, takes place at a very early period. At about the fourth week, in the human embryo, a septum begins to be formed in the ventricle; and by the end of the eighth week it is completed."

Dr. Dunglinson's observations are to the same effect. He says, "In the third month, the heart beats forcibly."

These authors clearly establish the fact of the vitality of the ovum as early as the seventeenth day of pregnancy, and thence onward, and consequently refute the idea of a "*communication*" of life at a subsequent period of utero gestation.

But, it may be asked, "is the fecundated ovum the very being to be hereafter developed?"

Let those who have investigated the subject answer.

"The Fœtus of three to four weeks, has the form of a serpent—its length from three to five lines; its head indicated by a swelling; caudal extremity (which is seen a white line, indicating the continuance of the medulla spinallis) slender, and terminating in the umbilical cord; the mouth indicated by a cleft; the members begin to appear as nipple-like protuberences; the line occupies the whole abdomen; the bladder is very large, &c."—*Carpenter.*

"From the time of the first evidence of impregnation to the fifteenth day, the product of conception appears only as a gelatinous, semi-transparent, floculent mass, of grayish color, liquifying promptly, and presenting no distinct formation, even by the aid of a microscope. At thirty days it has the size of a large ant, according to Aristotle, or of a barley-corn, according to Burton. Baudalocque, however, observes that it is not larger than the malleus of the tympanum. Its length varies from three to five lines.

"At six or seven weeks its length is almost ten lines. The orm and lineaments of the principal organs, and the places from which the members are to arise, can now be observed, and it is equal in size to a small bee.

"At this time, also, the fluid contained in the membranes, is much heavier than the embryo. At two months, the length is about two inches, and its weight nearly two ounces. All the parts are perfectly distinct, and many points of ossification, are observed in the head, trunk and members. Sometimes the male sex can be distinguished.

"At the third month it is about three and a half inches long, and between two and three ounces in weight. The nose and mouth are formed, and the features of the face become more distinct. The eyes are shut, and the eyelids adhere together—the head is longer, and heavier than the rest of the body—the umbilical cord is formed—the genitals are distinct—the penis, &c., are relatively very large—nympha are projecting, and the labia very thick.

"At the fourth month the fœtus is from five to six inches long, and weighs from four to five ounces. The external parts develop themselves, with the exception of the hair and nails. * * * During the fifth month the motions are felt by the mother. The length is from seven to nine inches, and the weight from nine to ten ounces. The brain is pulpy, and destitute of circumvolutions, or furrows, &c."—*Beck.*

"Haighton, in experimenting on rabbits, could not detect no change before the sixth day, and the fœtus was not perceptible until the tenth.

"In the case related by Sir Everard Home, to which we have so frequently referred, the embryo was perceptible under the microscope of Mr. Bauer, and although its weight did not probably exceed a grain, the future situation of the brain and spinal marrow was apparent. From this period, and especially after the fifteenth day, the ovule can be separated into two distinct sets of parts—the dependencies of the fœtus, and the fœtus itself. These, in the course of pregnancy, become more and more readily separable."

"From the moment of a fecundating copulation, the minute matters furnished, by both sexes, when commingled, commence the work of forming the embryo. For a short time, they find in the ovum the necessary nutriment, and subsequently obtain it from the uterus.

"The mode in which this action is accomplished is as mysterious as the essence of generation itself. Where the impregnated ovum is first seen, it seems to be an amorphous, gelatinous mass, in which no distinct organs are perceptible. In a short time, however, the brain and spinal marrow, and blood vessels make their appearance, but which of these is first developed is undecided.

"Sir Everard Home, from his observations of the chick in ovo, as well as from microscopic appearances presented by the ovum, in the case of the female who died on the seventh or eighth day after impregnation, in which a rudimental brain and spinal marrow were perceptible, decides that the parts first found, bear a resemblance to brain; and that the heart and arteries are produced in consequence of the brain having been established.

"The ovule does not reach the uterus until towards the termination of a week after conception. On the seventh or eighth day it has the appearance referred to, in the case so often cited from Sir Everard Home; the future situations of the brain and spinal marrow being recognisable with the aid of a powerful microscope. On the thirteenth or fourteenth day, according to Maygries, the ovum is perceptible in the uterus, and of about the size of a pea; containing a turbid fluid, in the midst of which an opaque point is suspended—*punctum saliens.* The weight of this has been valued at about a grain.

"On the twenty-first day, the embryo appears under the shape of a large ant, according to Aristotle, etc.

"Thus, man himself, in succession, passes through a great variety of forms, from the condition of a simple cell; these forms, merging by degrees into one another—the form of the serpent, of the fish, of the bird—and this not only as regards the entire system in the aggregate, but also as regards each one of its constituent mechanisms—the nervous system, the circulatory, the digestive. Now, on the passage onward, these forms are to be regarded, as has been well expressed—each one as the scaffolding by which the next is built—and just as man, in his embryonic transit, presents these successive aspects on a small scale, so does the entire animal series present them in the world, on the great scale. * * *

"Every living being, therefore, springs from a germ which will develop itself into the likeness of its parent, provided it is submitted to the same conditions through which its parents passed. * * * *

"During the development of any new organism, the new parts uniformly arise from the old ones; they are not built from foreign materials depositing themselves upon new centres, but are educed by the unfolding, enlarging and modeling of parts already existing. An organism is not developed as an enlarged house, by building part to part, but it all expands from one common or simple centre. * * * *

"At the stage we are now considering, the nutrition of the embryo is conducted in a special, but very temporary way. The yolk of the ovum has no stock of food to maintain the nutrition processes beyond the brief space which transpires in the passage through the fallopian tube. The duty of nutrition is, at this moment, assumed by the villous coat of the chorion, which absorbs fluid exuding from the uterine decidua, very much after the manner of the spongioles of the plant; but almost immediately the necessity arises of diverting more directly the albuminoid material to the quickly growing embryo from the yolk-bag, to which it would have gone, and this new destination implies the introduction of new channels of transport, which, under the form of a vascular apparatus, are now provided.

"About the close of the second month, a proper vascular apparatus, for the combined purposes of nutrition, secretion, and respiration, makes its appearance; it is the placenta. * *. From the description which has thus been given, it may be gathered, that up to the period of birth, three distinct types of nutrition have been followed. They may, with sufficient accuracy, be designated—1st, yolk nutrition; 2d, tuft nutrition; 3d, placental nutrition. To these, may be added the two followed at a later period—4th, lactation, and after the dental mechanism is supplied; 5th, the diet of mature life. * *.

"It has been shown, that at one period, nutrition is solely at the expense of the yolk of the ovum, which is appropriated by a simple surface—imbibition; and that this, in due time, is succeeded by what has been designated tuft nutrition. At a later period, this mode, in its turn, is replaced by another, depending on a vascular arrangement—the placenta. For a considerable period after birth, a fourth system is relied on—nourishment by milk; and it is only by degrees, when the necessary changes have been made in the digestive mechanism, the teeth being cut, that the final mode of nutrition is assumed."
—*Draper.*

Thus, from the united testimony of these distinguished authors, it is seen—first, that the identical ovum of fecundation, is that which, in its development, is the embryo of fifteen days, of thirty days, of two months, of three or four months, the fœtus in utero, at five months, at term of gestation, in parturition, through lactation, dentition, &c., &c. It is impossible to be mistaken at this point. But, secondly, it is as clearly shown that vitality is coëxistent with fecundation, and that there is, and, in the nature of the case, can be, no point of time afterwards for the *communication* of vitality. Thirdly, that the fecundated ovum is not only the embryonic man, already vital, but it is, in an important sense, an independent, self-existent being, that is having in itself the materials for development, being actually separated from the mother, as well as from the father, though maintaining a connexion in utero by the vascular arrangement repeatedly referred to; there is, *really*, as has been fully demonstrated, no actual attachment of the placenta to the uterus. Fourthly—that from the moment of fecundation, the ovum breaks from subjection to the influence of both parents, and instantly begins the work of its own development, which is to continue through a series of changes up to manhood, but not to the destruction of identity of being and attributes.

I ask, then, where, and how, shall the points of time be fixed, at which this being may, and may not, be destroyed? Is it not the same at every period of existence, in so far as it alone is considered? It is only in a relative point of view, that the murder of the adult differs from that of the fœtus in utero. As to the *nature* of the crime, there can be no difference in the sight of God.

But, I cannot consent to conclude this subject before introducing a further extract from the pen of Prof. Draper, who uses the following beautiful and strong language in summing up his views of this matter:

"Perhaps, in some age hereafter, Physiology will find herself sufficiently advanced to offer her opinion on this profound topic, (the immortality of the soul,) for I cannot think that God has left us without a witness in this matter, even in the structure and development of the body itself. From the moment that we see the first traces of the nervous mechanism lying in the primitive groove, we recognise the subordination of every other part to that mechanism.

"For it, and because of it, are introduced the digestive, the circulatory, the secretory, the respiratory apparatus. They are merely its ministers. And fastening our attention on the course which it pursues, we see that it is at once a course of concentration and development.

"The special, is at each instant, coming out of the more general, and from the beginning to the end, psychical development. The germinal membrane is cast away as soon as a stomach can be prepared—aquatic respiration ceases as soon as aerial can be maintained. The scaffolding that was of use at one moment, is thrown aside as soon as a new elevation is reached. The germ, the embryo, the infant, are only successive points in a progress, which at instant displays, this casting away the means that have been used, as soon as they are done with. That is the style in which the work is carried on. The principle which obscurely animated the germ, is the same which, in a higher way, animates the embryo, and this again, is the same which, in a more exalted condition, animates the infant and the man. The cloudy speck which ushers in the phantasmagoria of life, expands, as the Great Artist directs, until every lineament has become visible.

"That active Agent, which was first laid in a fold of the germinal membrane, was not annihilated when its type of life was changed to placental, and, therefore, aquatic life. It withstood the shock, when again, after a due season, it was suddenly made to breathe the air. Arrived at the mature condition, there is not in its companion body, a single particle that was present at birth. All has changed. And, what is still more important, not only has there been this interstitial removal, but in succession, the very nature of every one of its organs has changed. It is needless now to repeat how many different systems of nutrition it has depended on—how many sorts of stomachs in succession it has had—how it has carried on its circulation with a heart; with a heart of one cavity, and finally with one of four. Through, all these losses and changes, the unmaterial principle has passed unscathed, and even gathering strength.

"In the broadest manner that a fact can be set forth, we see, herein, the complete subordination of structure, and the enduring character of spirit."

